# Genomic and pathological insights into the first identified genotype IIIb chicken anemia virus strain in Bangladesh

**DOI:** 10.1128/spectrum.00796-25

**Published:** 2025-10-10

**Authors:** Marjana Akter, Roni Mia, S.M. Nazmul Hasan, Anandha Mozumder, Nurejunnati Jeba, Raduyan Farazi, Farzana Akter, Sharmin Akter, Md. Zaminur Rahman, Sukumar Saha, Tofazzal Islam, Md. Golzar Hossain

**Affiliations:** 1 Department of Microbiology and Hygiene, Bangladesh Agricultural University 54492, Mymensingh, Bangladesh; 2 Department of Physiology, Bangladesh Agricultural University 54492, Mymensingh, Bangladesh; 3 Institute of Biotechnology and Genetic Engineering, Gazipur Agricultural University 198780, Gazipur, Bangladesh; Taichung Veterans General Hospital, Taichung, Taiwan, Province of China

**Keywords:** chicken anemia virus, genotype IIIb, whole-genome sequencing, phylogenetics, VP3 mutations

## Abstract

Chicken anemia virus (CAV) is a highly infectious pathogen that causes severe immunosuppression and significant economic losses in poultry, with limited genomic data in Bangladesh hindering effective disease control. This study characterizes a CAV strain from a field outbreak in Bangladesh through clinical, pathological, and molecular analyses, including genome sequencing, genotyping, and assessment of viral protein structure. Clinically suspected chickens exhibiting anemia, depression, pale combs, and cyanotic wings underwent gross and histopathological examinations. Viral DNA was extracted from bone marrow samples and analyzed using PCR, followed by complete genome sequencing with next-generation sequencing. Phylogenetic analysis, genotyping, mutational profiling, and structural predictions of viral proteins (VP1, VP2, and VP3) were performed to assess evolutionary relationships and pathogenic potential. Histopathology confirmed severe lymphoid depletion in the thymus, spleen, and bursa of Fabricius, consistent with CAV-induced immunosuppression. Molecular detection confirmed CAV presence in eight out of ten samples. Whole-genome analysis revealed a 2,330 bp genome with 42% GC content, clustering within genotype IIIb and showing close genetic relatedness to a Chinese strain. Mutational analysis revealed several nucleotide substitutions in the viral genomes, with the highest number of amino acid changes observed in the VP3 protein. Computational modeling revealed minor structural variations in VP1 and VP2, which may affect antigenicity and phosphatase activity. This study provides the complete genome characterization of a Bangladeshi CAV strain, revealing critical genetic variations that may influence viral virulence. The findings underscore the need for enhanced surveillance and targeted vaccine strategies to mitigate CAV-related losses in poultry.

**IMPORTANCE:**

This study provides the first complete genomic characterization of a genotype IIIb chicken anemia virus (CAV) strain in Bangladesh. By integrating clinical, pathological, and molecular analyses, the research identifies critical genetic variations that could influence viral virulence, immune evasion, and disease severity. The findings highlight the close genetic relationship between this Bangladeshi strain and a previously reported Chinese strain, suggesting potential epidemiological links. Furthermore, the study underscores the need for enhanced surveillance and targeted vaccine strategies to mitigate CAV-induced immunosuppression and economic losses in poultry farming. The insights gained from this research contribute to a deeper understanding of CAV evolution and could inform future diagnostic and control measures to protect poultry populations.

## INTRODUCTION

Chicken infectious anemia, caused by the chicken anemia virus (CAV), poses a significant economic threat to the global poultry industry, including Bangladesh (1). CAV primarily infects chicks between 2 and 4 weeks of age, leading to severe pathological changes such as anemia, hemorrhagic syndrome, blue wing disease, bone marrow atrophy, and thymic atrophy (2). However, its most critical impact is immunosuppression, as the virus invades hematopoietic cells in the bone marrow and T lymphoblasts in the thymus. This results in higher mortality rates and increased susceptibility to secondary infections, including Newcastle disease, Marek’s disease, and various pathogenic bacteria and fungi (3, 4). CAV can be transmitted both horizontally and vertically, with its virulence depending on factors such as virus strain, dose, and transmission route (5, 6). While chickens of all ages are susceptible, clinical disease primarily manifests in young chickens (2–4 weeks). In contrast, older birds typically develop subclinical infections with minimal clinical signs but can serve as virus reservoirs (7). Morbidity and mortality rates can reach as high as 80% and 55% in susceptible young chickens (5, 8).

CAV is a non-enveloped virus with icosahedral symmetry, classified under the genus *Gyrovirus* in the *Anelloviridae* family (9). Its genome consists of a single-stranded, closed-circular DNA, approximately 2.3 kb in length. The genome contains three major overlapping open reading frames encoding three viral proteins: one structural protein (VP1) and two non-structural proteins (VP2 and VP3) (10). VP1, the major capsid protein, is crucial for viral replication, transmission, and virulence, as it induces host-neutralizing antibodies. The non-structural protein VP2 acts as a dual-specific protein phosphatase and serves as a scaffolding protein that ensures the proper conformation of VP1 during viral assembly (11). VP3, also known as apoptin, induces apoptosis in chicken thymocytes, T lymphoblastoid cells, and myeloid cells (12).

CAV was first identified in Japan in 1979 and has since been circulating globally (13). In Bangladesh, it was first reported in 2002 across multiple districts, but studies on circulating strains remain limited (14). Due to its immunosuppressive nature, CAV-infected chickens often suffer from secondary infections, stunted growth, and poor feed conversion, leading to substantial economic losses for farmers and increased costs for consumers (15). Furthermore, controlling CAV is challenging due to the complexities of developing effective prevention strategies and the high costs associated with treatment. Understanding the molecular epidemiology, evolutionary patterns, and genetic variability of circulating CAV strains is essential for mitigating the disease’s devastating effects. This knowledge can facilitate vaccine development and improve diagnostic methods. Therefore, in this study, we identified CAV from a field outbreak through clinical symptoms, postmortem findings, and PCR. Histopathological lesions in the spleen, thymus, bursa of Fabricius, liver, lungs, and heart were examined. The complete viral genome was sequenced, followed by phylogenetic analysis, genotyping, mutation detection, and structural analysis of major viral proteins.

## MATERIALS AND METHODS

### Sample collection

Ten (10) chickens exhibiting typical symptoms of CAV infection were collected and transported to the Department of Microbiology and Hygiene at Bangladesh Agricultural University. Chicken samples were collected from a commercial broiler farm in Narsingdi district, central Bangladesh, experiencing an acute outbreak with ~10% morbidity but no recorded mortality. The affected flock comprised 23-day-old Hubbard cross broilers derived from a parent breeder flock. The farm operated an all-in-all-out system with a capacity of 2,550 birds, and no CAV vaccination program had been implemented at either the breeder or broiler level. Additionally, two healthy birds of the same age were collected as controls. The birds were dissected, and samples from the liver, bone marrow, spleen, bursa of Fabricius, heart, lungs, and thymus were collected for further experiments, including gross and microscopic examination and viral inoculum preparation.

### Inoculum preparation

Five grams of each bone marrow sample was collected, finely minced using sterile scissors and forceps, and homogenized in a mortar with sterile sand using a pestle (16). The homogenate was suspended in phosphate-buffered saline to prepare a 10% (wt/vol) solution. This suspension underwent three freeze-thaw cycles to facilitate cell disruption, followed by two centrifugation cycles at 3,000 rpm for 10 minutes each. The supernatant was collected and treated with antibiotics and antimycotic agents to prevent contamination. The processed samples were then used for DNA extraction and subsequent molecular virus detection.

### Gross and histopathological investigation

The thymus, spleen, bursa of Fabricius, lungs, liver, and heart were examined for gross pathological changes and submitted to the Histopathology Laboratory in the Department of Surgery and Obstetrics at Bangladesh Agricultural University for detailed histopathological analysis. Each tissue sample was dissected, fixed in 10% formalin, embedded in paraffin wax, and processed using standard histological protocols, including hematoxylin and eosin staining (17). The stained sections were examined microscopically under an OLYMPUS CX41 microscope, and high-resolution photomicrographs were captured at the Department of Physiology, Bangladesh Agricultural University, Mymensingh, for further evaluation and documentation.

### DNA extraction and PCR amplification

DNA extraction from the prepared inoculum was performed using the TIANamp Virus DNA/RNA Kit (China), following the manufacturer’s instructions. The extracted DNA was either immediately used for PCR or stored at −20°C for later use. The partial VP1 gene was amplified using PCR with the following primer set: VP1-F: 5′-ATG GCA AGA CGA GCT CGC-3′ and VP1-R: 5′-TCA GGG CTG CGT CCC CCA-3′, producing a 1,350 bp fragment (18). PCR amplification was performed in a 20 µL reaction mixture containing 1.0 µL (500 nM final concentration) of each forward and reverse primer, 6 µL of DNA template, 10 µL of Master Mix (TaKaRa Taq Version 2.0 plus dye), and 2 µL of double-distilled water. The reaction conditions were as follows: initial denaturation at 94°C for 5 minutes, 35 cycles of denaturation at 94°C for 1 minute, annealing at 59°C for 1 minute, and extension at 72°C for 1.5 minutes, a final extension at 72°C for 10 minutes, followed by storage at 4°C. For amplification of the partial VP2 gene, the primer set CAV-1 (5′-CTA AGA TCT GCA ACT GCG GA-3′) and CAV-2 (5′-CCT TGG AAG CGG ATA GTC AT-3′) was used to generate a 419 bp DNA fragment, following a previously published protocol (19). PCR-amplified products were separated on a 1.5% agarose gel prepared in 1 × TBE buffer and stained with ethidium bromide. Electrophoresis was performed at 100 V for 120 minutes, and the amplified products were visualized and analyzed using a GelDoc Go system (BioRad, USA).

### Whole-genome sequencing

The complete CAV viral genome was sequenced using next-generation sequencing (NGS) technology (Illumina NovaSeq 6000). The sequencing methodology was previously reported (20). Briefly, genomic DNA libraries were prepared using the Rapid Plus DNA Lib Prep Kit for Illumina (Cat# RK20208). Sequencing reads underwent quality assessment (FastQC v0.11.9), adapter and low-quality base trimming (Trimmomatic v0.39), and host DNA removal. Filtered reads were aligned to the reference genome of chicken anemia virus isolate CAVDL21 (NCBI accession no. OQ749509.1) using BWA (21). Circular genome confirmation was achieved through *de novo* assembly (Unicycler) and annotation with Prokka (v1.14.6) (22). The final assembly showed ~300 × coverage with overlapping termini, confirming circularity. The complete genome, designated CAV/Narsingdi-1-MGH-BD, has been deposited in the NCBI databases.

### Phylogenetic tree construction and genotyping

A total of 38 whole-genome sequences of CAV strains were retrieved from GenBank using NCBI BLAST. Multiple sequence alignment of the reference sequences and CAV/Narsingdi-1-MGH-BD was performed using MEGA11 software. A phylogenetic tree was constructed using pairwise distance matrices and the neighbor-joining method (23). The percentage of replicate trees in which the associated taxa clustered together in the bootstrap test (1,000 replicates) was annotated next to the branches, indicating confidence levels of inferred clusters (24). Genotyping was conducted following a previously published protocol (25). Complete coding regions of the VP1 gene of various CAV genotypes were retrieved from GenBank and used for genotyping.

### Mutational analysis and protein structure prediction

Reference sequences (accession nos.NC_001427.1 and AF395114.1) were retrieved from the NCBI database (https://www.ncbi.nlm.nih.gov/). Nucleotide substitutions in the *VP1, VP2*, and *VP3* gene sequences of CAV/Narsingdi-1-MGH-BD were aligned with the retrieved reference sequences using CLC Sequence Viewer (Version 8.0). To identify amino acid substitutions, the translated sequences of VP1, VP2, and VP3 of CAV/Narsingdi-1-MGH-BD were aligned with the reference sequences using CLC Sequence Viewer. The tertiary structures of VP1, VP2, and VP3 proteins were predicted using the Swiss-Model online server (https://swissmodel.expasy.org/) with default parameters. Protein sequences were submitted to the server, and models were built based on the highest identity and GMQE values. The structures were visualized using PyMOL software and validated using Procheck tools from SAVESv6.0 (https://saves.mbi.ucla.edu/) and the ProSA-web server (https://prosa.services.came.sbg.ac.at/prosa.php) by analyzing the Ramachandran plot and Z-score, respectively.

## RESULTS

### Clinical, gross, and histopathological findings

**Fig 1:**
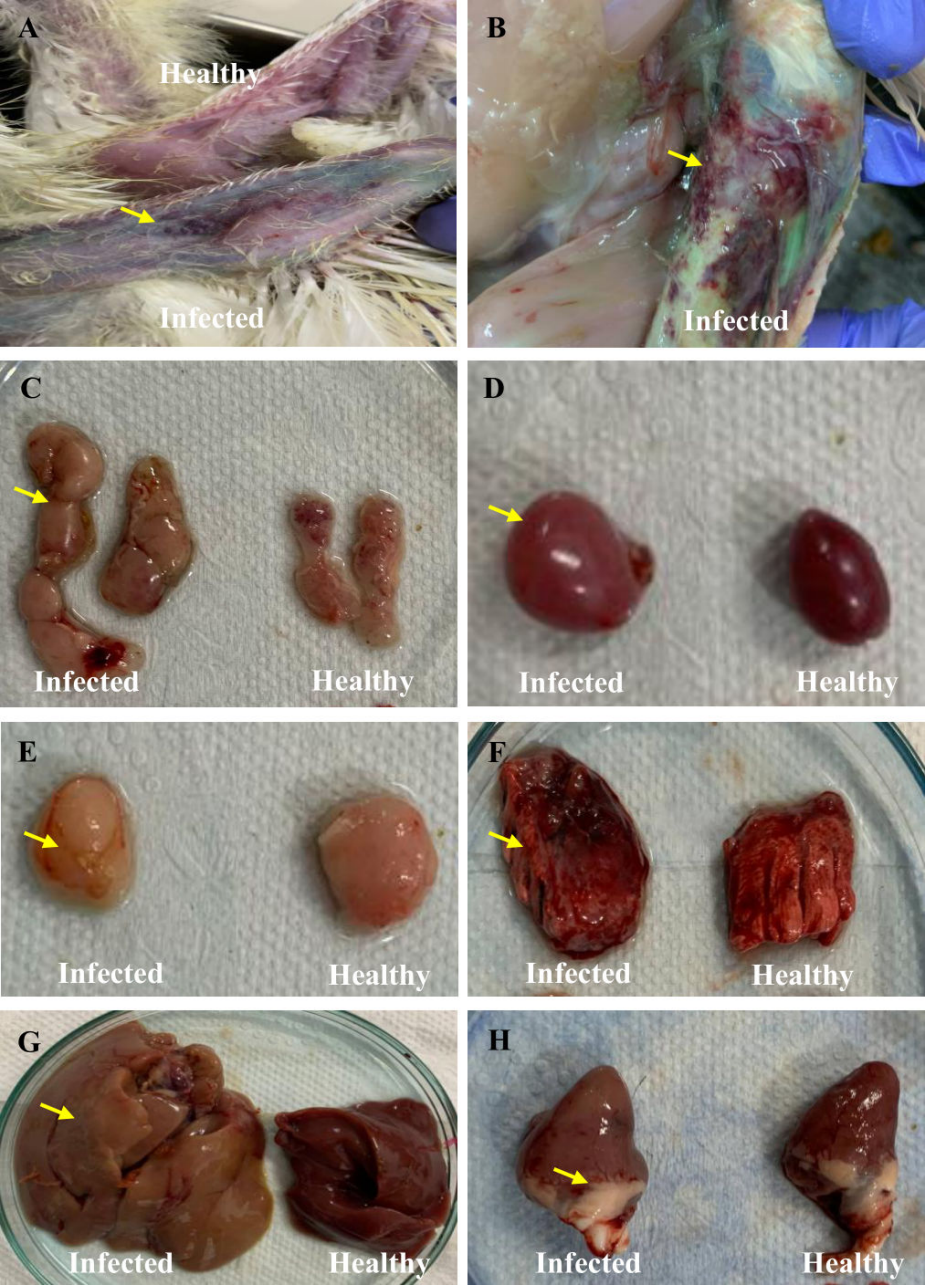
Gross pathological findings of the CAV-infected chickens. (**A**) Cyanotic wing. (**B**) Subcutaneous hemorrhage. (**C**) Hypertrophic thymus. (**D**) The swollen spleen. (**E**) Pale and atrophied bursa. (**F**) Hemorrhagic lung. (**G**) Pale and hypertrophic liver. (**H**) Hemorrhagic lesions in the heart. Yellow arrows indicate the lesions in the mentioned organs of the CAV-infected chickens.

**Fig 2:**
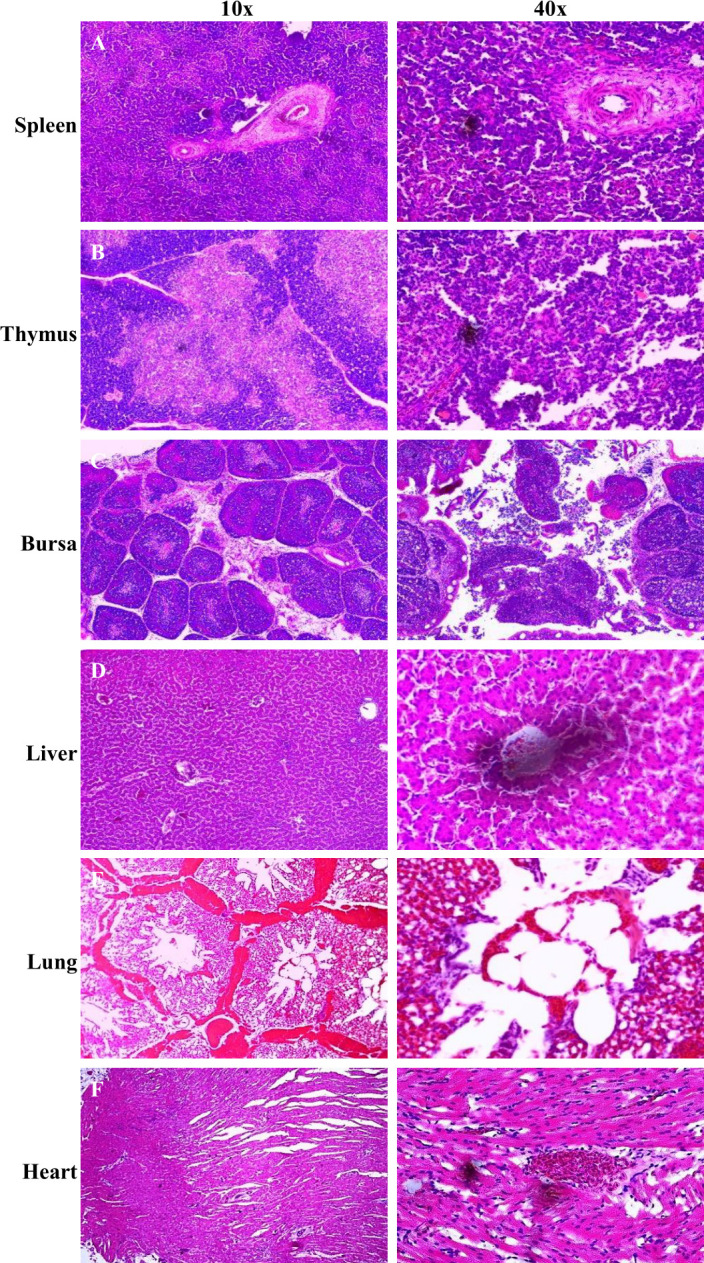
Histopathological alterations in the different organs of the CAV-infected chickens. (**A**) Lymphoid depletion and necrosis of the spleen. (**B**) Cortical lymphocyte depletion and absence of demarcation between the medulla and cortex of the lobe of the thymus. (**C**) Lymphocyte depletion in the bursa of Fabricius. (**D**) Liver showing necrosis and degeneration. (**E**) Congestion and hemorrhage in the lungs. (**F**) Necrosis and degeneration of myocardial tissue in the heart. Yellow arrows indicate the lesions in histopathological sections of the mentioned organs of the CAV-infected chickens.

The affected chickens showed typical clinical symptoms of CAV infection, including anemia, depression, pale combs, and blue or cyanotic wings (Fig. 1A). The morbidity rate of the chickens was recorded at approximately 10%, as documented in the farm’s record book. Gross pathological examinations revealed significant changes in the infected chicks compared to healthy ones. The infected chicks displayed hemorrhage in the subcutaneous layer, lungs, and scattered hemorrhagic spots in the heart (Fig. 1B, F, and H). A pale and slightly atrophic bursa of Fabricius was observed (Fig. 1E). Additionally, the infected chicks exhibited hypertrophic thymuses and pale-colored, swollen spleens and livers, whereas healthy chickens showed no such lesions (Fig. 1C, D and E). Histopathological examinations revealed massive lymphoid depletion in the spleen, thymus, and bursa (Fig. 2A, B and C). Necrosis in the spleen, cortical thinning of the thymus, and atrophy with structural disorganization of bursal follicles were observed (Fig. 2A, B and C). Necrosis and degeneration of hepatocytes in the liver and myocardial tissue in the heart were noted (Fig. 2D and F). The lungs exhibited severe congestion and hemorrhage (Fig. 2E).

### Molecular detection and whole-genome sequencing

Viral DNA extracted from the bone marrow of suspected CAV-infected and healthy chickens was analyzed using gene-specific primers for the *VP1* and *VP2* genes. Gel electrophoresis and subsequent UV transillumination confirmed that eight out of ten bone marrow samples exhibited target-specific bands of 1,350 bp and 419 bp, respectively. As expected, no viral DNA bands were detected in the bone marrow of healthy chickens. One PCR-positive sample, exhibiting strong VP1 (1,350 bp) and VP2 (419 bp) bands indicative of high viral load, was selected for whole-genome sequencing using NGS; the corresponding chicken displayed typical clinical signs and severe histopathological lesions of CAV. The complete CAV genome was 2,330 bp with 42% GC content and contained three coding regions encoding VP1 (1,350 bp), VP2 (651 bp), and VP3 (366 bp), consistent with the complete CAV genomes previously reported in the literature (2,298–2,319 bp) (10). The complete genome sequence of the CAV isolate (CAV/Narsingdi-1-MGH-BD) has been assigned the GenBank, BioSample, and SRA accession nos. PQ412955, SAMN44002588, and PRJNA1167429, respectively (20).

### Phylogenetic analysis and genotypic characterization

**Fig 3:**
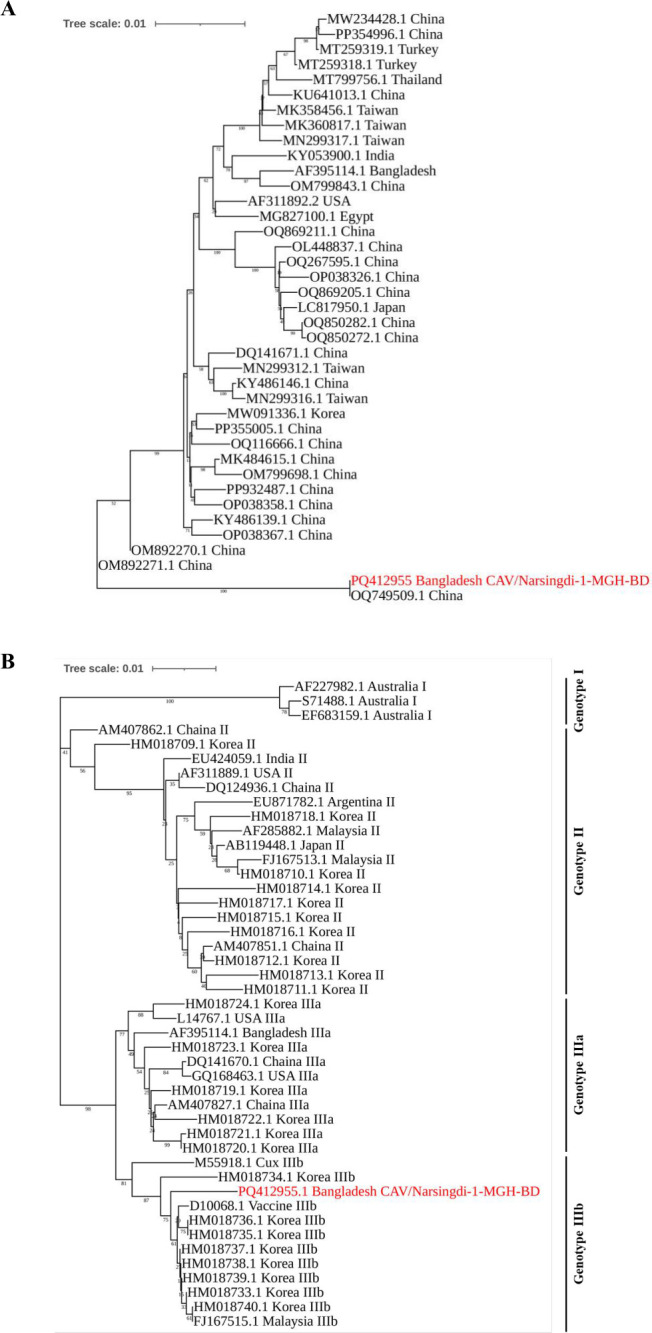
Phylogenetic analysis and genotyping of the identified CAV genome. (**A**) Phylogenetic analysis. Phylogenetic tree constructed using the complete genome sequence of the identified CAV strain (CAV/Narsingdi-1-MGH-BD) and 38 reference strains from GenBank. The tree was generated using the neighbor-joining (NJ) methods with the maximum composite likelihood model and 1,000 bootstrap replicates. The tree is scaled, with branch lengths measured in the number of substitutions per site (next to the branches). (**B**). Genotype determination of the identified strain based on the *VP1* gene. Complete *VP1* gene sequences of various genotypes of the CAV were extracted from the GenBank, and a tree was generated using NJ methods with 1,000 bootstrap replicates. The CAV strain identified in this study is marked with red color.

A phylogenetic tree was constructed based on multiple sequence alignments of the complete nucleotide sequence of CAV/Narsingdi-1-MGH-BD and 37 reference strains retrieved from GenBank. The tree indicated that the identified CAV strain was closely related to the Chinese strain (OQ749509.1) and formed an independent cluster (Fig. 3A). Genotyping based on VP1 gene sequences identified CAV/Narsingdi-1-MGH-BD as genotype IIIb, clustering within the CAV genotype IIIb group (Fig. 3B).

### Mutational analysis of CAV/Narsingdi-1-MGH-BD compared to reference strains

**Fig 4:**
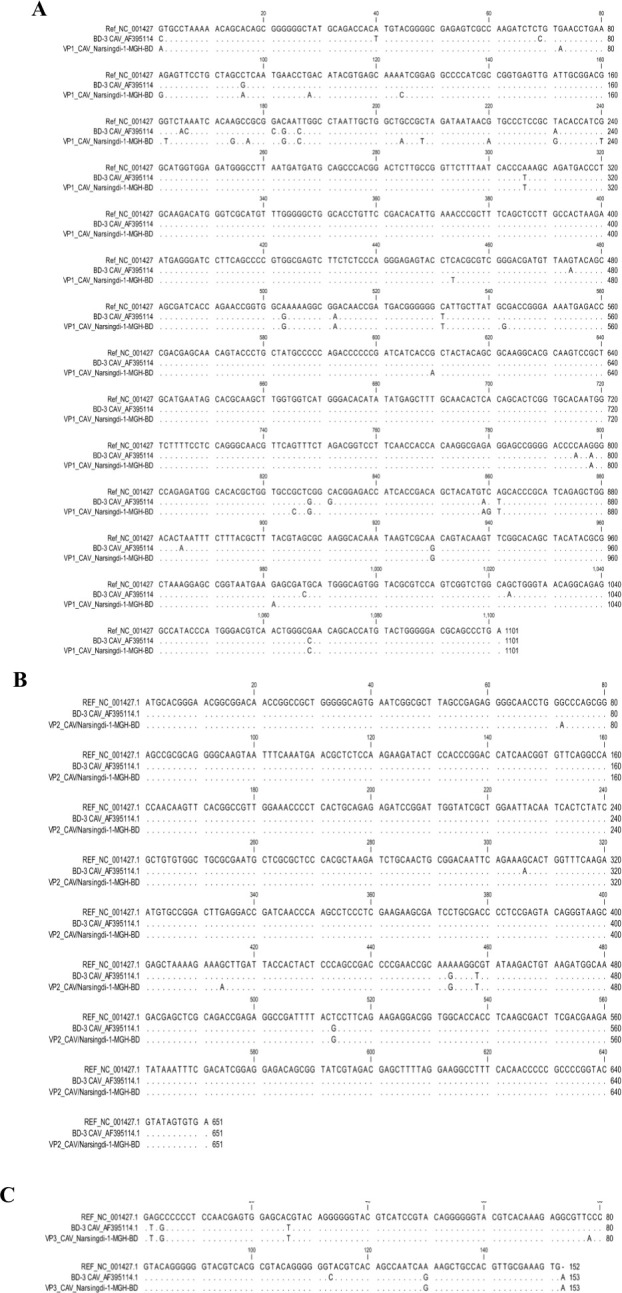
Nucleotide substitution analysis of viral proteins. Nucleotide substitution analysis of (**A**) *VP1* gene, (**B**) *VP2* gene, and (**C**) *VP3* gene of the CAV/Narsingdi-1-MGH-BD, aligning with two reference strains (NC_001427 and AF395114) using CLC viewer 8.0 software. Dots indicate identical nucleotides.

**Fig 5:**
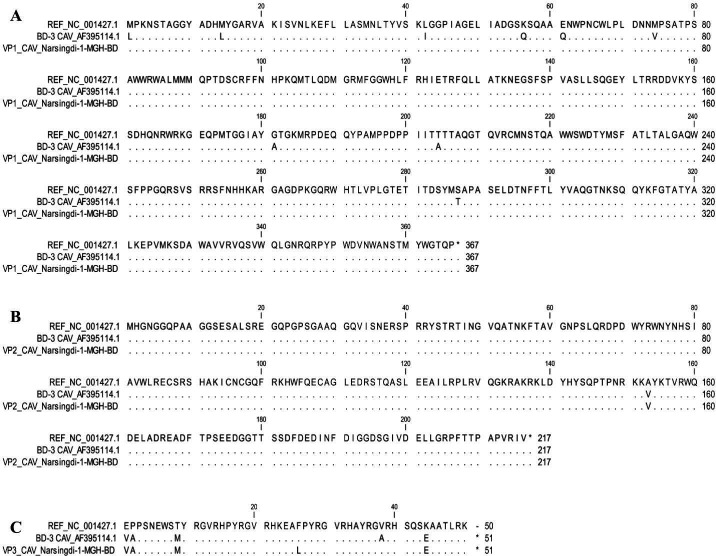
Mutational analysis of viral proteins. Amino acid substitution analysis of (**A**) VP1, (**B**) VP2, and (**C**) VP3 proteins of the CAV/Narsingdi-1-MGH-BD, aligning with two reference strains (NC_001427 and AF395114) using CLC viewer 8.0 software. Dots indicate identical residues.

Mutational analysis of the complete genome was performed in comparison with the NCBI reference strain and BD-3, the only previously reported complete CAV genome from Bangladesh, identified in 2004. Various synonymous and nonsynonymous nucleotide substitutions were detected in the *VP1*, *VP2*, and *VP3* genes of CAV/Narsingdi-1-MGH-BD compared to both the NCBI reference strain and BD-3 (Fig. 4A through C). For VP1, multiple nucleotide substitutions were identified (Fig. 4A), but these were all synonymous mutations compared with the NCBI reference strain (NC_001427.1), resulting in no amino acid changes. However, comparison with BD-3 revealed nine nonsynonymous mutations resulting in amino acid substitutions (Fig. 5A). For VP2, nucleotide variations were identified that resulted in a single nonsynonymous mutation (A153V) in comparisons with the reference strain and BD-3 (Fig. 5B). VP3 showed the highest rate of nonsynonymous mutations with five amino acid substitutions (E1V, P2A, T9M, F26L, and K44E) compared to the NCBI reference strain, while only two nonsynonymous changes (F26L and A38V) were observed relative to BD-3 (Fig. 5C).

### Physicochemical properties and predicted tertiary structures of viral proteins

**Fig 6:**
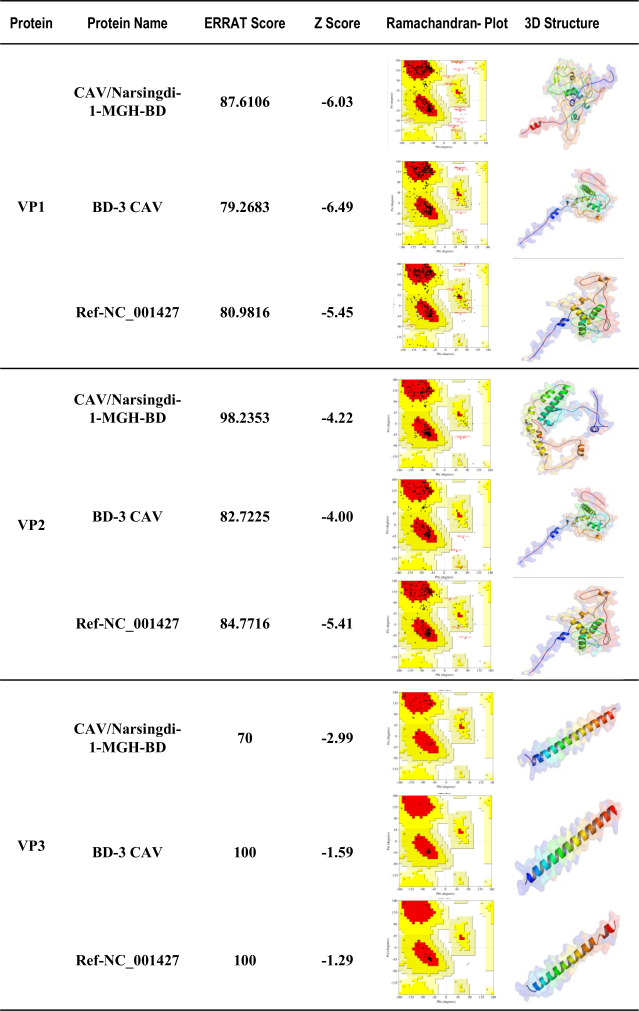
Prediction and tertiary structures analyses of the viral proteins. Structural predictions of VP1, VP2, and VP3 proteins of CAV/Narsingdi-1-MGH-BD using Swiss-model. Predicted 3D structures with ERRAT scores, Z-scores, and Ramachandran plots demonstrating structural stability and validation. Distribution of amino acid residues in Ramachandran plots, highlighting conformational differences due to amino acid substitutions.

The ProtParam program was used to characterize the physicochemical properties of the viral proteins, including the number of amino acids, molecular weight (MW), isoelectric point (pI), instability index (II), aliphatic index (AI), and grand average of hydropathicity (GRAVY). The findings indicated slight variations in the VP1, VP2, and VP3 proteins of CAV/Narsingdi-1-MGH-BD compared to reference sequences, except for VP3, which exhibited greater stability (Table 1). Comparative analysis of amino acid sequences between CAV/Narsingdi-1-MGH-BD and BD-3 revealed significant differences that may affect protein function. The nine amino acid substitutions in VP1 compared to BD-3 alter the physicochemical properties, with BD-3 showing a higher AI (57.92 vs 54.31), suggesting differences in thermostability. The single A153V mutation in VP2 is particularly significant as valine substitution introduces a more hydrophobic residue that may affect the protein’s phosphatase activity. For VP3, the two mutations (F26L and A38V) compared to BD-3 result in dramatic differences in protein stability, with BD-3 VP3 showing an II of 145.4 compared to 42.68 in our strain (Table 1), indicating our strain’s VP3 is significantly more stable. The Ramachandran plot analysis (Table 3) shows that our strain’s VP3 has only 66.90% of residues in the most favorable region compared to 100% for BD-3, suggesting conformational differences that may affect apoptin function. The predicted tertiary structures of VP1, VP2, and VP3 proteins in CAV/Narsingdi-1-MGH-BD displayed minor structural differences compared to reference strains due to amino acid substitutions (Fig. 6, Tables 2 and 3).

**TABLE 1:** Physiochemical properties of VP1, VP2, and VP3 proteins

Protein	Strain	II	pI	Theoretical pI	GRAVY score	MW, kDa
VP1	CAV/Narsingdi-1-MGH-BD	50.8	54.31	9.38	−0.596	41.47
	BD-3 CAV_AF395114	49.95	57.92	9.4	−0.561	41.30
	Ref-NC_001427	50.38	54.45	9.38	−0.594	41.37
VP2	CAV/Narsingdi-1-MGH-BD	60.85	54.6	7.7	−1.11	24.14
	BD-3 CAV_AF395114	60.68	54.35	11.28	−0.853	23.50
	Ref-NC_001427	60.84	52.28	11.03	−0.817	24.64
VP3	CAV/Narsingdi-1-MGH-BD	42.68	54.60	11.24	−1.11	5.87
	BD-3 CAV_AF395114	145.4	35.53	12.67	−1.934	6.23
	Ref-NC_001427	140.73	35.32	12.34	−2.147	6.33

**TABLE 2:** Properties of secondary structure of VP1, VP2, and VP3 proteins

Protein	Strain	Alpha helix (%)	Extended strand (%)	Random coil (%)
VP1	CAV/Narsingdi-1-MGH-BD	21.31	10.11	68.58
	BD-3 CAV_AF395114	20.77	10.66	68.58
	Ref-NC_001427	21.31	10.11	68.58
VP2	CAV/Narsingdi-1-MGH-BD	25.93	7.87	66.22
	BD-3 CAV_AF395114	13.04	13.04	73.91
	Ref-NC_001427	20.55	9.59	69.86
VP3	CAV/Narsingdi-1-MGH-BD	22.00	8.00	70.00
	BD-3 CAV_AF395114	72.34	4.26	23.40
	Ref-NC_001427	48.94	8.51	42.55

**TABLE 3:** Amino acid residues of VP1, VP2, and VP3 proteins in Ramachandran plot

Protein	Strain	Most favorable region (%)	Additional allowed region (%)	Generously allowed region (%)	Disallowed region (%)
VP1	CAV/Narsingdi-1-MGH-BD	82.40	12.50	1.90	3.20
	BD-3 CAV_AF395114	82.10	16.00	0.60	1.30
	Ref-NC_001427	80.40	17.30	1.30	1.00
VP2	CAV/Narsingdi-1-MGH-BD	88.40	10.50	0.00	1.10
	BD-3 CAV_AF395114	83.30	15.10	1.10	0.50
	Ref-NC_001427	83.30	15.10	1.15	0.50
VP3	CAV/Narsingdi-1-MGH-BD	66.90	31.70	2.40	0.00
	BD-3 CAV_AF395114	100	0.00	0.00	0.00
	Ref-NC_001427	100	0.00	0.00	0.00

## DISCUSSION

CAV is a significant pathogen affecting poultry, primarily targeting hematopoietic and immune system components (7, 26, 27). This study presents the clinical, pathological, and molecular characterization of an identified CAV strain, including complete genome sequencing, genotyping, and structural analyses of viral proteins. These findings underscore the mechanisms of viral infection and the genomic evolution of CAV within the Bangladeshi poultry population.

CAV primarily infects hemocytoblasts in the bone marrow and precursor T cells in the thymus, resulting in aplastic anemia and immunosuppression (28). Clinical signs such as anemia, depression, pale comb, and cyanotic wings indicate severe hematopoietic disruption due to viral replication in bone marrow cells. CAV typically induces anemia and immunosuppression in young chickens, characterized by pallor, atrophy of lymphoid organs (thymus, spleen, and Bursa of Fabricius), and a swollen, mottled liver (29). However, in this study, hypertrophic pallor spleens and thymuses were observed atypically. Concurrent infections with other pathogens, such as fowl adenoviruses, can alter the typical disease progression of CAV by immunosuppression (30). Although CAV is classified as a single serotype, genotyping remains epidemiologically and clinically relevant for several reasons. Different genotypes exhibit varying levels of pathogenicity; for example, genotype III strains have been associated with more severe clinical disease, even in the presence of maternal antibodies (25, 31, 32). A Chinese study further demonstrated that mortality rates can vary significantly among different CAV strains under experimental conditions (33). Experimental studies have demonstrated that genotype III CAV infections can cause swollen, pale livers and spleens in infected chicks (34). Additionally, hemorrhagic lungs and hearts observed in CAV-infected chickens in this study suggest the possibility of concurrent infections, although specific pathogens were not investigated. Variations in the virulence of CAV strains could also contribute to differing pathological outcomes (34). The identification of genotype IIIb in our study, reported for the first time in Bangladesh and distinct from the previously described genotype II (BD-3), highlights the need to evaluate whether current vaccine strains provide optimal protection against circulating field viruses. Despite the availability of vaccines, the high seroprevalence reported in Bangladesh (14) suggests that vaccination alone may not be sufficient for effective CAV control. Our detection of a genotype IIIb strain with unique mutations, particularly in the stable VP3 protein, underscores the importance of integrating vaccination strategies with stringent biosecurity measures.

Histopathological examinations confirmed significant lymphoid depletion in the thymus, bursa of Fabricius, and spleen, indicating impaired T-cell maturation and reduced immune competence (29). The VP3 protein, also known as apoptin, plays a crucial role in inducing apoptosis in hematopoietic precursor cells, leading to reduced erythropoiesis and subsequent anemia (35, 36). Apoptin-mediated apoptosis is triggered via the mitochondrial pathway, interacting with cellular apoptotic regulators such as Bcl-2, caspase-9, and caspase-3 (37). The observed histopathological changes in the spleen and thymus are consistent with apoptosis-mediated lymphoid depletion, confirming the immunosuppressive nature of CAV infection.

The molecular detection of viral DNA in the bone marrow of infected chickens confirms the hematopoietic tropism of CAV. Complete genome sequencing of CAV/Narsingdi-1-MGH-BD revealed a genome length of 2,330 bp, which is distinctly different from the previously identified BD-3 strain (2,298 bp) from Bangladesh. The 2,330 bp genome size falls within the typical range for complete CAV genomes (2,298–2,319 bp) (10). Variations in reported CAV genome sizes primarily result from differences in non-coding regulatory regions, particularly the region between the polyadenylation signal and the start of VP1. Despite these differences, both CAV/Narsingdi-1-MGH-BD and BD-3 encode the three key structural proteins—VP1, VP2, and VP3. Phylogenetic analysis indicated that CAV/Narsingdi-1-MGH-BD is closely related to a Chinese strain (OQ749509.1) and clusters within genotype IIIb, whereas BD-3 belongs to genotype II (25). This finding suggests a potential epidemiological link between Bangladeshi and Chinese CAV strains, likely facilitated by poultry trade and movement. The genotypic classification further suggests the introduction of new CAV strains into Bangladesh or ongoing viral evolution over the past two decades.

Mutational analysis revealed several synonymous and nonsynonymous nucleotide substitutions in viral genes, along with amino acid changes in viral proteins, compared to both the NCBI reference strain and BD-3, the only previously sequenced CAV strain from Bangladesh (38). The nine amino acid differences in VP1 between our strain and BD-3 represent substantial genetic divergence within Bangladeshi CAV populations. These mutations may indicate adaptation to local conditions or the introduction of divergent strains. Notably, our strain shows no VP1 amino acid changes compared with the NCBI reference strain, whereas BD-3 exhibits multiple differences, suggesting that our strain may be closer to the ancestral CAV lineage in terms of VP1 sequence. In addition, computational analysis of physicochemical properties indicated that the VP1, VP2, and VP3 proteins of CAV/Narsingdi-1-MGH-BD exhibit minor variations in MW, pI, and hydropathicity compared to reference strains. The marked difference in VP3 stability between our strain (II 42.68) and BD-3 (145.4) is particularly noteworthy. VP3 (apoptin) plays a key role in inducing apoptosis in infected cells, and the higher stability observed in our strain may contribute to more efficient apoptosis induction and potentially greater pathogenicity (39). Predicted tertiary structures of VP1, VP2, and VP3 revealed slight conformational differences, likely influenced by amino acid substitutions. Structural variations in VP1 may affect antigenicity and immune recognition, while changes in VP2 could alter its phosphatase activity, potentially impacting immune modulation (10, 11, 29). The VP3 mutations may enhance apoptotic efficiency, exacerbating immunosuppression and disease severity in infected chickens (29, 35).

In conclusion, this study highlights the clinical, pathological, and molecular characteristics of a newly identified CAV strain in Bangladesh. The findings confirm that CAV/Narsingdi-1-MGH-BD exhibits significant genetic variation compared to previously reported strains. Phylogenetic analysis establishes its close relation to a Chinese strain, placing it within genotype IIIb. The observed mutations, particularly in VP3, may have implications for viral pathogenesis and immune evasion. Further investigation is needed to conduct large-scale epidemiological studies and assess the functional impact of these genetic variations on virulence and vaccine efficacy. These findings contribute to a deeper understanding of CAV evolution and its epidemiological significance in poultry health management.
